# Long-Term Effects of Peripubertal Binge EtOH Exposure on Hippocampal microRNA Expression in the Rat

**DOI:** 10.1371/journal.pone.0083166

**Published:** 2014-01-08

**Authors:** Sarah A. Prins, Magdalena M. Przybycien-Szymanska, Yathindar S. Rao, Toni R. Pak

**Affiliations:** Loyola University Chicago Health Science Division, Department of Cell and Molecular Physiology, Maywood, Illinois, United States of America; Kent State University, United States of America

## Abstract

Adolescent binge alcohol abuse induces long-term changes in gene expression, which impacts the physiological stress response and memory formation, two functions mediated in part by the ventral (VH) and dorsal (DH) hippocampus. microRNAs (miRs) are small RNAs that play an important role in gene regulation and are potential mediators of long-term changes in gene expression. Two genes important for regulating hippocampal functions include brain-derived neurotrophic factor (BDNF) and sirtuin-1 (SIRT1), which we identified as putative gene targets of miR-10a-5p, miR-26a, miR-103, miR-495. The purpose of this study was to quantify miR-10a-5p, miR-26a, miR-103, miR-495 expression levels in the dorsal and ventral hippocampus of male Wistar rats during normal pubertal development and then assess the effects of repeated binge-EtOH exposure. In addition, we measured the effects of binge EtOH-exposure on hippocampal Drosha and Dicer mRNA levels, as well as the putative miR target genes, BDNF and SIRT1. Overall, mid/peri-pubertal binge EtOH exposure altered the normal expression patterns of all miRs tested in an age- and brain region-dependent manner and this effect persisted for up to 30 days post-EtOH exposure. Moreover, our data revealed that mid/peri-pubertal binge EtOH exposure significantly affected miR biosynthetic processing enzymes, Drosha and Dicer. Finally, EtOH-induced significant changes in the expression of a subset of miRs, which correlated with changes in the expression of their predicted target genes. Taken together, these data demonstrate that EtOH exposure during pubertal development has long-term effects on miRNA expression in the rat hippocampus.

## Introduction

Heavy episodic alcohol consumption (i.e. binge drinking) has been steadily increasing among adolescents in recent decades [1], [2]. Indeed, data from the Department of Health and Human Services: Substance Abuse and Mental Health services Administration (2005) showed that an alarming 90% of the alcohol consumed by youth occurs in a binge-like pattern, defined as raising the blood alcohol concentration (BAC) above the legal driving limit (0.08%) within a 2 hour time period (NIAAA, 2012). Extensive remodeling of the brain occurs during adolescence, which includes changes in cortical gray and white matter, synaptic connectivity, and increased neurogenesis [3], [4], [5], [6], [7] and alcohol exposure during this critical time can have severe detrimental effects on brain function [8], [9], [10], [11], [12], [13]. Studies from our laboratory and others have demonstrated that adolescent binge-pattern alcohol exposure results in long-term dysregulation of the neuroendocrine stress response, memory impairments and behavioral deficits [14], [15], [16], [17], [18]. An altered stress response often underlies depression- and anxiety-related disorders and importantly, these conditions are commonly experienced by over 50% of alcohol-dependent patients [15], [19], [20]. Indeed, mood and memory impairments are often present in tandem with alcohol abuse and neuropsychiatric illnesses that emerge post-puberty [21], [22].

The hippocampus is an important brain region that mediates learning, memory, and mood and it has been well established that hippocampus structure and function is impaired by EtOH abuse [23], [24], [25], [26], [27], [28], [29]. Notably, pubertal EtOH abuse inhibits adult neurogenesis, impairs learning and memory in adulthood, and impairs information retention. The precise molecular mechanisms mediating the long-term effects of adolescent binge EtOH exposure are poorly understood, however short non-coding regulatory RNAs are sensitive to EtOH and have recently been recognized as critical mediators of nearly every basic cellular process [30], [31]. In particular, microRNAs (miRs, ∼22 nucleotide single-stranded non-coding RNA) regulate the translation of proteins important for neuronal development during embryogenesis, postnatal neuronal maintenance and survival, and hippocampal neurogenesis throughout life [32], [33], [34], [35], [36], [37]. Moreover, disruption of mature miR expression and/or function has been linked to alcohol-induced neurological afflictions including addiction and fetal alcohol spectrum disorder (FASD) [38], [39]. In this study we used a Wistar rat model to identify EtOH-sensitive miRs that target genes involved in regulating hippocampal processes, such as memory and mood. Using multiple target prediction algorithms (Targetscan: www.targetscan.org; miR database: www.miRDB.org) [40], [41], [42], we identified brain-derived neurotrophic factor (BDNF) as a target gene of miR-10a-5p, miR-26a, miR-103 and miR-495, and sirtuin 1 (SIRT1) as a target gene of miR-26a, miR-103 and miR-495. BDNF is a major regulator of synaptic plasticity and thought to be aberrantly regulated in psychiatric disorders, and SIRT1 is a class III protein deacetylase that has been recently associated with anxiety behavior [19], [20], [43].

We tested the hypothesis that mid/peripubertal binge EtOH exposure alters hippocampal miR expression and that this leads to changes in the expression of their target genes. Importantly, the normal expression profile of these particular miRs during pubertal development has not been reported in any species studied to date. Therefore, we first quantified the normal developmental expression profile of miR-10a-5p, miR-26a, miR-103 and miR-495 in the rat hippocampus at three time points in pubertal development (early, mid/peri, and late). Next, we determined how mid/peri-pubertal binge EtOH exposure altered those normal expression levels immediately following EtOH exposure, as well as 30 days after the last EtOH exposure. Gene expression levels of miR processing enzymes, Drosha and Dicer, were also quantified at each time point in order to determine a possible molecular mechanism for EtOH effects. Finally, the putative downstream target genes BDNF and SIRT1 were quantified at each time point and correlated with the changes in miR expression. Overall, our data provide evidence that mid/peripubertal binge EtOH exposure induces long-term alterations in mature miR expression levels in the rat hippocampus, which has the potential to modulate the expression of their downstream target genes.

## Methods

### Animals

**Figure 1:**
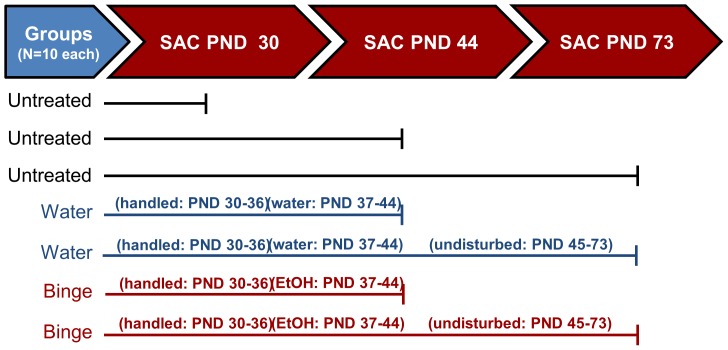
Diagram of experimental paradigm. Diagram depicting the age of sacrifice and specific treatment paradigms for each group of male Wistar rats. N = 10/group.

Male Wistar rats were purchased from Charles River Laboratories (Wilmington, MA) at weaning (postnatal day (PND) 23) and allowed to acclimate for 7 days, after which they were randomly distributed into 3 groups: early pubertal (PND 30), mid/peri-pubertal (PND 37), and late pubertal (PND 73, Fig. 1). The early, peri-pubertal and late pubertal age animals each had an untreated group (N = 10/age group) which were sacrificed at PND 30, PND 44 and PND 73, respectively. In addition to the untreated groups at each age, there were 2 groups administered treatments at mid/peri-pubertal age (N = 20/treatment group; total of 40 animals): peri-pubertal water (control) and peri-pubertal binge EtOH treated. The water and EtOH groups were handled for 5 minutes once/day beginning at PND 30 to eliminate non-specific effects of handling stress (Fig. 1). Mid/peri-pubertal water or EtOH treatments (see methods below) began on PND 37, an age which has been previously defined as peri-puberty based on circulating gonadotropin levels and stages of spermatogenesis [44], [45], [46]. Half of the mid/peri-pubertal water/EtOH treated animals were sacrificed 60 min. following the last EtOH/water treatment at PND 44 (N = 10 water +N = 10 EtOH). The remaining animals (N = 10 water +N = 10 EtOH) were left undisturbed following the last EtOH treatment in their home cage until sacrificed at late puberty (PND 73). All animals were pair-housed on a 12∶12 light/dark cycle with lights on at 07.00 h. Food and water were available *ad libitum*.

### Ethics statement

All animal protocols were approved by the Institutional Animal Care and Use Committee at Loyola University Chicago permit #2011002. All measures were taken to minimize animal numbers and suffering.

### Binge exposure paradigm and treatment design

Mid/peri-pubertal (PND 37) animals were randomly assigned to either 1) peripubertal water (N = 20) or 2) peri-pubertal binge EtOH (N = 20) treatment groups (Fig. 1). These animals were in addition to the untreated groups (N = 10) at each age, such that a total of 70 animals were used in these studies. The animals in the binge EtOH group received EtOH treatment (3 g/kg, 20% v/v in water) via oral gavage every morning at 10:00 AM once/day for 3 consecutive days, followed by 2 days of water only, and then an additional 3 days of EtOH (i.e. a total of 6 EtOH treatments over the course of 8 days). This binge exposure paradigm has been used previously to mimic the pattern of binge alcohol consumption typically observed in adolescents [12], [47]. Moreover, our previous studies have shown that this repeated binge-pattern EtOH paradigm did not affect body weight/growth curves during pubertal development and consistently resulted in blood alcohol concentrations (BAC) of 150–180 mg/dl in males [12], [47], [48]. The water group was administered room temperature tap water via oral gavage once/day for 8 consecutive days. The animals were euthanized by rapid decapitation 60 minutes following EtOH treatment (PND 44, “immediate EtOH effects”) or 30 days following the last day of treatment at late puberty (PND 73, “long-term EtOH effects”). The blood alcohol concentration in EtOH treated animals was 172±12.3 mg/dl, which is consistent with our previous reports using this peri-pubertal binge EtOH paradigm [12]. It is important to note that the untreated and the water-treated mid/peri-pubertal groups were not statistically different for any parameter measured and were therefore, combined into one group for further statistical analyses.

### Tissue collection

Trunk blood and brains were collected immediately following decapitation. Trunk blood was collected on ice into heparinized glass tubes, centrifuged at 4000 rpm for 10 minutes, plasma separated and stored at −20°C until processed for testosterone levels using enzyme immunoassay (EIA, see below). Brains were rapidly dissected, flash-frozen in isopentane (−35°C) on dry ice, and stored at −80°C until further processing. Frozen brains were sectioned at 200 µm on a freezing microtome, mounted onto glass slides, and the ventral and dorsal hippocampi were microdissected using a 0.75 mm Palkovit's brain punch tool (Stoelting Co., Wood Dale, IL). The specificity of the microdissected regions was confirmed using The Rat Brain in Stereotaxic Coordinates, Fourth Edition Atlas (G. Paxinos and C. Watson). The ventral hippocampus was defined as between 1.8 mm to 3.8 mm posterior to Bregma, 3 mm below the top of the brain and 6.6 mm from the bottom of the brain. The dorsal hippocampus was defined as between 4.16 mm and 6.05 mm posterior to Bregma, 3 mm below the top of the brain and 2 mm above the bottom of the brain. Brain tissue punch samples were collected on ice into microcentrifuge tubes containing 1 ml of Trizol reagent (Invitrogen, Inc., Carlsbad, CA). Tissue samples were sonicated on ice prior to total RNA isolation.

### RNA isolation

Total RNA was isolated from tissue punch samples using Trizol reagent (Invitrogen Inc., Carlsbad, CA) according to the manufacturer's directions. All RNA samples were analyzed for quality by Nanodrop spectrophotometry and visualization of the RNA on a 1.5% agarose gel.

### Quantitative reverse transcription PCR (qRT-PCR)

Following RNA isolation, 1.0 µg total RNA was reverse transcribed using the First Strand Synthesis SuperMix for qRT-PCR (Invitrogen, Inc., Carlsbad, CA) for mRNA detection, and 1.0 µg total RNA was used for NCode miRNA First-Strand cDNA Synthesis Kit for miRNA detection. miRNA and mRNA qRT-PCR was performed with Fast Start Universal SYBR Green Master Mix (Roche) on an Eppendorf Realplex4 with a silver block. Forward primers for specific miRNAs were designed as described in the Ncode™ miRNA First-Strand cDNA synthesis kit handbook (Invitrogen, Inc., Carlsbad, CA) and using miRBase 18 as a sequence reference. The small RNA, U6 and housekeeping gene hypoxanthine guanine phosphoribosyl transferase 1 (HPRT) were used as a loading control and to normalize the data for miRNA and mRNA analysis, respectively, as neither were altered by EtOH treatment [12]. The following thermocycler program was used for mRNA target genes: 1) 95°C for 10 minutes, 2) 95°C for 30 seconds, 3) 59°C for 30 seconds, 4) 72°C for 30 seconds, and melting curve analysis. The following thermocycler program was used for miRNA: 1) 95°C for 10 minutes, 2) 95°C for 30 seconds, 3) 65.3°C for 20 seconds, 4) 72°C for 12 seconds. Quantification of the gene expression was achieved using the ΔΔCt method [49]. The following intron-spanning primers were used for analysis of selected miRNA target genes and for miRNA processing enzymes:

SIRT1:5′GCGGCCGCGGATAGGTCCATA, 3′TCCCACAGGAGACAGAAACCCCA, BDNF: 5′AGCCTCCTCTGCTCTTTCTGCTGGA, 3′CTTTTGTCTATGCCCCTGCAGCCTT,
Drosha: 5′GAAGTCACCGTGGAGCTGAGTA, 3′ATCATTGCATGCTGACAGACATC,
Dicer: 5′GGGAAAGTCTGCAGAACAAAC AND 3′GGCTGTCTGAGCTCTTAGTTC.

The following forward primers were used for analysis of selected mature miRNA along with a universal reverse primer provided in the NCode miRNA First-Strand cDNA Synthesis Kit:

miR-10a-5p: 5′CGCTACCCTGTAGATCCGAATTTGTG,
miR-26a: 5′CCGGGTTCAAGTAATCCAGGATAGGC,
miR-103: 5′GGAGCAGCATTGTACAGGGCTATGA,
miR-495: 5′CGCGAAACAAACATGGTGCACTTCTT.

### Hormone measurements

Plasma levels of testosterone were measured using a commercially available EIA kit (Cayman Chemical, Ann Arbor, MI) according to manufacturer's instructions. The range of detection was between 3.9 and 500 pg/ml and the intra-assay CVs was 2.2%. Briefly, plasma samples were combined with testosterone acetylcholinesterase (AChE) tracer and testosterone EIA antiserum and incubated in a 96-well IgG-coated plate for 2 hr. at room temperature. Samples were washed 5 times with provided wash buffer then combined with Ellman's reagent containing the substrate for AChE, and the plate was developed for 60 minutes, shaking and covered, at room temperature. Absorbance was read at 420 nm on a multimode Synergy HT plate reader (BioTek Instruments, Inc., Winooski, VT).

### Blood alcohol concentration assay

Trunk blood samples were collected into heparinized tubes, centrifuged at 3000 rpm for 10 min at 4°C; and plasma stored at −20°C. Blood alcohol levels were determined by measuring the change in absorbance at 340 nm following enzymatic oxidation of EtOH to acetylaldehyde (Point Scientific Alcohol Reagent Kit). Assay range is 0 to 400 mg/dl and intra and interassay CV = 6.4% and 7.9%, respectively.

### Statistical analysis

Statistical analyses were performed by the Biostatistics Core Facility at Loyola University Stritch School of Medicine in consultation with Dr. James Sinacore. Data obtained from qRT-PCR was analyzed by two-way Analysis of Variance (ANOVA) followed by Tukey's posthoc test for all pair-wise comparisons when there was a significant main effect and interaction. All tests were performed using SigmaStat Statistical Analysis Software. A p-value of less than 0.05 was designated as significant. Exclusion criteria comprised of outliers greater than or equal to 3 times the standard deviation of the mean.

## Results

### Mature miR-10a-5p, miR-26a, and miR-495 expression levels in the dorsal hippocampus of untreated male rats are age dependent

**Figure 2:**
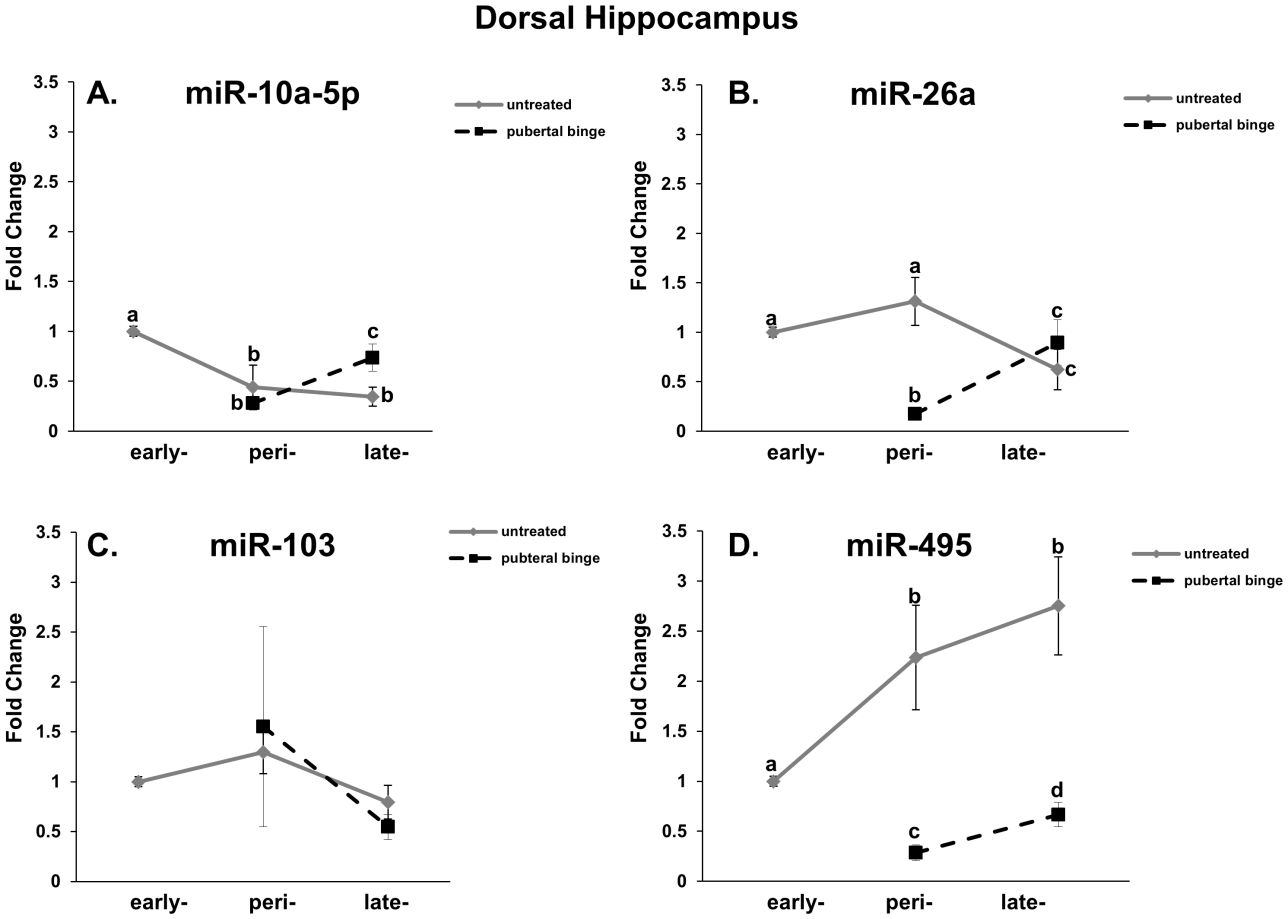
Peri-pubertal binge EtOH exposure alters miR expression during pubertal development in the dorsal hippocampus. miR-10a-5p (A), miR-26a (B), miR-103 (C), and miR-495 (D) expression levels in untreated (solid line) and EtOH-treated (dashed line) pubertal male rats. N = 10/group. Data represent mean fold change ±SEM as compared to untreated PND 30 animals. Dissimilar letters indicate a statistically significant difference between groups (p<0.05).

Expression levels of these specific mature miRs in the brain during pubertal development have not been previously described. Therefore, we first determined the normal developmental profile of mature miR-10a-5p, miR-26a, miR-103 and miR-495 expression in the dorsal hippocampus using untreated male Wistar rats. Mature miR expression levels were measured using qRT-PCR at three time points throughout pubertal development (early  = 30 d, mid/peri  = 44 d, late  = 73 d). Importantly, water-treated animals at mid/peri-puberty were not statistically different from untreated animals at that same age for any parameter tested in either brain region, therefore the two groups (untreated and water-treated) were combined for purposes of statistical analyses. In the dorsal hippocampus, a two-way ANOVA revealed a main effect of age on the expression levels of all miRs tested, except miR-103 (Table 1). Each of the three miRs that showed a significant effect of age in the dorsal hippocampus had a distinct developmental pattern. For instance, miR-10a-5p expression decreased significantly between early and mid/peri-puberty, and remained lower than early puberty levels until late puberty (Fig. 2A, solid line). By contrast, miR-26a expression did not change between early and mid/peri-puberty, but significantly decreased at late puberty (Fig. 2B, solid line). Finally, a significant increase was observed in miR-495 expression between early and mid/peri-puberty and these levels remained high until late puberty (Fig. 2D, solid line).

**Table 1:** Statistical Analysis of Gene Expression Levels in the Dorsal Hippocampus.

miRNA	MAIN EFFECT OF AGE	MAIN EFFECT OF TREATMENT	INTERACTION: AGE X TREATMENT
miR-10a-5p	Yes: F(2,33) = 12.293. p<0.001	No	No
miR-26a	Yes: F(2,44) = 3.222. p<0.049	Yes: F(1,44) = 5.212. p<0.027	Yes: p<0.001
miR-103	No	No	No
miR-495	Yes: F(2,37) = 4.923. p<0.013	Yes: F(1,37) = 59.23. p<0.001	Yes: p<0.001

GENE	MAIN EFFECT OF AGE	MAIN EFFECT OF TREATMENT	INTERACTION: AGE X TREATMENT
Drosha	Yes: F(2,54) = 24.906. p<0.001	No	Yes: p = 0.008
Dicer	Yes: F(2,54) = 18.725. p<0.001	No	Yes: p<0.001
BDNF	Yes: F(2,52) = 12.845. p<0.001	Yes: F(1,52) = 6.574. p<0.013	No
SIRT1	No	Yes: F(1,54) = 110.941. p<0.001	Yes: p<0.001

### Binge EtOH exposure during mid/peri-puberty significantly alters normal developmental profile of miRs in the dorsal hippocampus

Next, we determined the effects of repeated binge EtOH exposure during mid/peri-puberty on these miR levels in the brain. Rats were administered our repeated binge-pattern EtOH exposure paradigm (see methods) and dorsal hippocampal miR expression of miR-10a-5p, miR-26a, miR-103 and miR-495 was compared with untreated rats/water-treated rats immediately following binge EtOH exposure and one-month post-EtOH exposure. Our results demonstrated a significant main effect of EtOH treatment on the expression of miR-26a and miR-495 and there was also a significant interaction between age and treatment, demonstrating that the effects of EtOH were age dependent (Table 1). Although there was not a significant main effect of EtOH treatment on miR10a-5p levels, they were significantly higher by late puberty in the EtOH treated group compared to controls (Fig. 2A). miR-26a was significantly decreased compared control animals immediately following binge EtOH exposure at mid/peri-puberty, but this difference did not persist and was equivalent to those of untreated animals by late puberty (Fig. 2B). miR-103 was not significantly altered by mid/peri-pubertal EtOH treatment in the dorsal hippocampus, similar to the results observed with age alone (Fig. 2C). Most striking were the results of EtOH exposure on miR-495. Similar to miR-26a, miR-495 was significantly decreased as a result of binge EtOH exposure at mid/peri-puberty. Notably however, expression levels remained significantly below normal even one-month post EtOH exposure (Fig. 2D), suggesting a potential long-term effect of pubertal binge EtOH exposure on miR-495 in the dorsal hippocampus.

### Mature miR-10a-5p, miR-26a, miR-103, and miR-495 expression levels in the ventral hippocampus of untreated male rats are age-dependent

**Figure 3:**
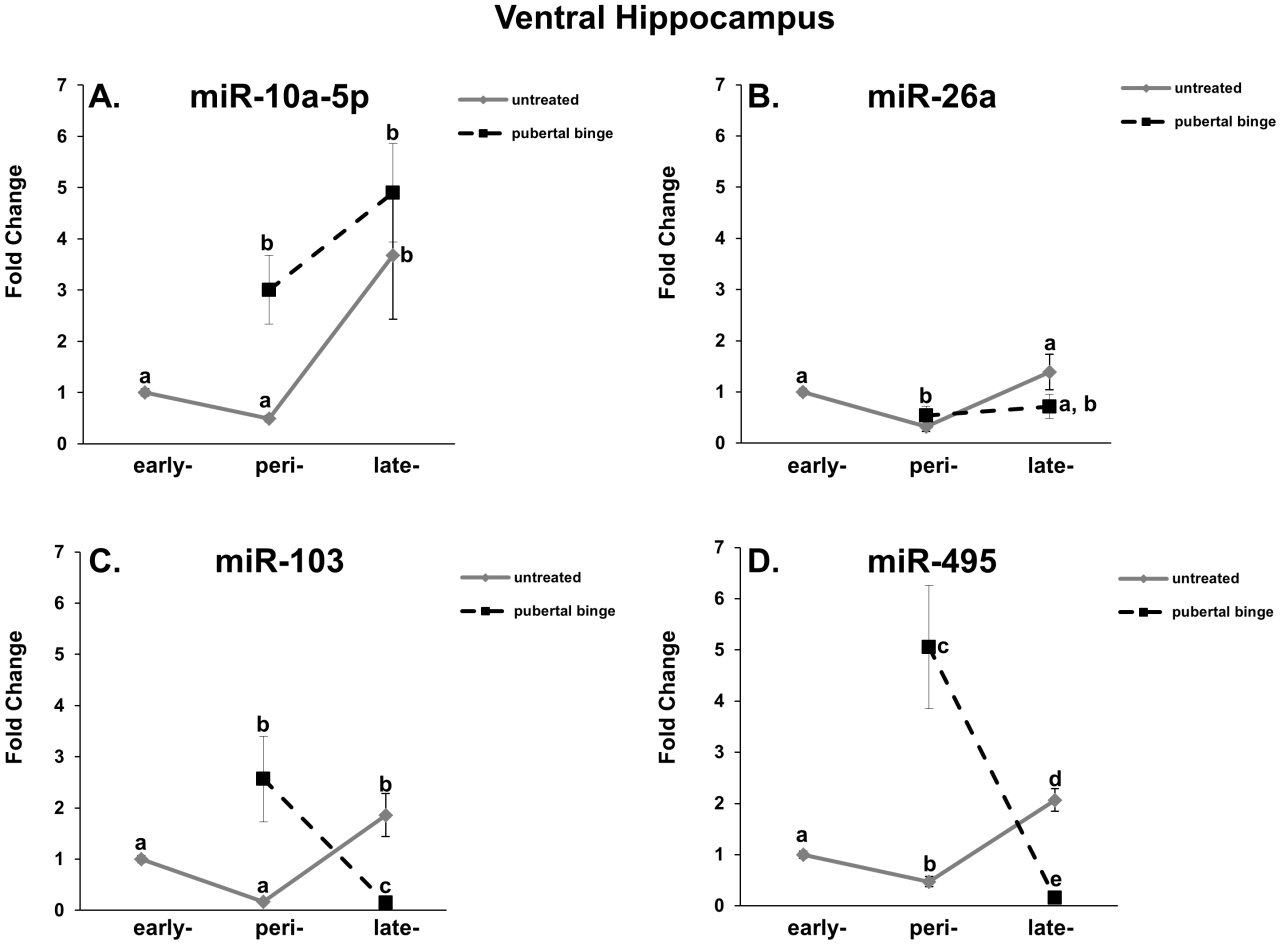
Peri-pubertal binge EtOH alters miR expression during pubertal development in the ventral hippocampus. miR-10a-5p (A), miR-26a (B), miR-103 (C), and miR-495 (D) expression levels in untreated (solid line) and EtOH-treated (dashed line) pubertal male rats. N = 10/group. Data represent mean fold change ±SEM as compared to untreated PND 30 animals. Dissimilar letters indicate a statistically significant difference between groups (p<0.05).

Distinct region and age-dependent expression of miRs has been demonstrated in the brain of a variety of species [50], [51], [52]. Therefore, we quantified the expression of miR-10a-5p, miR-26a, miR-103, and miR-495 in the ventral hippocampus across pubertal development in untreated rats to determine if there were region specific miR expression patterns in the hippocampus. In the ventral hippocampus, there was a significant main effect of age in the untreated animals on all four miRs tested, including miR-103, which previously did not show a significant change across pubertal development in the dorsal hippocampus (Table 2). Specifically, miR10a-5p showed no change between early and mid/peri-puberty, but significantly increased by late puberty in the ventral hippocampus (Fig. 3A, solid line). Also, in contrast to the dorsal hippocampus miR-26a significantly decreased at mid/peri-puberty, but this change did not persist and was equivalent to early pubertal levels by late puberty (Fig. 3B, solid line). The ventral hippocampus levels of miR-103 and miR-495 had a similar profile. Both had a statistically significant decrease, or strong trend towards decrease, at mid/peri-puberty compared to early pubertal levels, but then the levels increased significantly above that of early pubertal levels by late puberty (Fig. 3C, D, solid line). Notably, miR-495 expression in the ventral hippocampus demonstrated the most dynamic expression profile throughout pubertal development, as it had distinct expression levels at each time point measured.

**Table 2:** Statistical Analysis of Gene Expression Levels in the ventral hippocampus.

miRNA	MAIN EFFECT OF AGE	MAIN EFFECT OF TREATMENT	INTERACTION: AGE X TREATMENT
miR-10a-5p	Yes: F(2,42) = 17.492. p<0.001	Yes: F(1,42) = 8.847. p = 0.005	No
miR-26a	Yes: F(2,37) = 5.064. p = 0.011	No	No
miR-103	Yes: F(2,54) = 4.582. p = 0.015	Yes: F(1,54) = 5.739. p = 0.02	Yes: p<0.001
miR-495	Yes: F(2,42) = 8.359. p<0.001	Yes: F(1,42) = 5.998. p = 0.019	Yes: p<0.001

GENE	MAIN EFFECT OF AGE	MAIN EFFECT OF TREATMENT	INTERACTION: AGE X TREATMENT
Drosha	Yes: F(2,54) = 4.650. p = 0.014	No	Yes: p<0.001
Dicer	Yes: F(2,54) = 10.746. p<0.001	No	Yes: p<0.001
BDNF	Yes: F(2,41) = 13.622. p<0.001	Yes: F(1,41) = 9.109. p = 0.004	Yes: p<0.001
SIRT1	Yes: F(2,43) = 16.484. p<0.001	Yes: F(1,43) = 14.759. p<0.001	Yes: p<0.001

### Repeated adolescent binge EtOH exposure differentially alters expression of miR-10a-5p, miR-26a, miR-103 and miR-495 in the ventral hippocampus

We predicted that mid/peri-pubertal binge EtOH exposure would alter the normal developmental profile of miR expression in the ventral hippocampus, based on the evidence obtained from the dorsal hippocampus. Indeed, there was a significant main effect of treatment and a significant interaction between age/treatment for miR-10a-5p, miR-103, and miR-495 in the ventral hippocampus (Table 2). Interestingly, the magnitude of changes in miR expression was overall much higher in the ventral hippocampus compared to the dorsal, with some miRs changing by as much as 5-fold (Fig. 3). One example of a large fold change was observed with miR-10a-5p. The mature expression levels of miR-10a-5p were significantly increased by an average of 3–5 fold following mid/peri-pubertal binge EtOH exposure (Fig. 3A, dashed line). Further, the expression levels continued to show an increase at late puberty, paralleling the untreated group at that same age (Fig. 3A). There was no observed statistical effect of mid/peri-pubertal EtOH treatment on miR-26a. Nevertheless, the expression levels in the EtOH-treated group did not appear to follow the normal age-dependent increase observed by late puberty (Fig. 3B). The most striking effects of mid/peri-pubertal binge EtOH exposure in the ventral hippocampus were observed in miR-103 and miR-495 expression, as their normal developmental expression levels at both mid/peri-puberty and late-puberty were opposite following mid/peri-pubertal binge EtOH exposure. Overall, our results demonstrate both immediate and long-term effects of mid/peri-pubertal binge EtOH exposure in the rat hippocampus and these effects were distinct between the dorsal and ventral regions (Table 3).

**Table 3:** Summary of mid/peri-pubertal EtOH exposure on miRNA and mRNA gene expression.

miRNA	Dorsal Hipp immediate ETOH effect	Dorsal Hipp long-term ETOH effect	Ventral Hipp immediate ETOH effect	Ventral Hipp long-term ETOH effect
10a-5p	−	↑	↑	−
26a	↑	−	−	−
103	−	−	−	↓
495	↓	↓	↓	↓

Gene	Dorsal Hipp immediate ETOH effect	Dorsal Hipp long-term ETOH effect	Ventral Hipp immediate ETOH effect	Ventral Hipp long-term ETOH effect
Drosha	↑	↑	↑	↓
Dicer	↑	↓	↑	↓
BDNF	↑	↑	↓	−
SIRT1	↑	↑	↓	−

Arrows indicate a statistically significant effect (increase/decrease) of EtOH compared to age-matched water-treated controls.

### Mature miR biosynthetic processing enzymes are altered by mid/peri-pubertal binge EtOH exposure in the dorsal and ventral hippocampus

**Figure 4:**
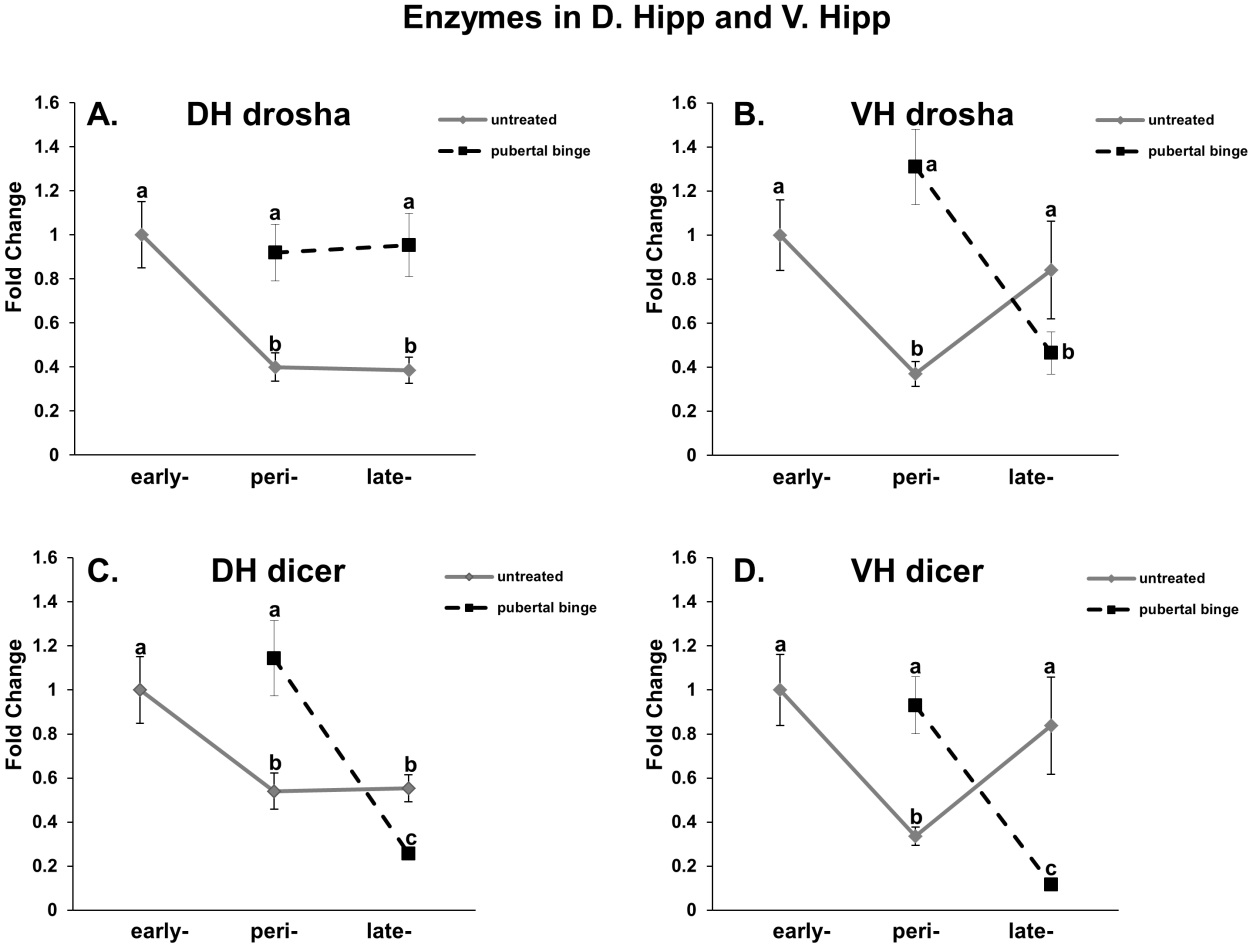
Peri-pubertal binge EtOH exposure differentially alters miR biosynthetic processing enzymes in the dorsal and ventral hippocampus. Drosha (A, C) and Dicer (B, D) mRNA levels in the dorsal and ventral hippocampus in untreated (solid line) and EtOH-treated (dashed line) pubertal male rats. N = 10/group. Data represent mean fold change ±SEM as compared to untreated PND 30 animals. Dissimilar letters indicate a statistically significant difference between groups (p<0.05).

Primary miR transcripts are transcribed from the genome in a RNA polymerase II dependent manner and sequentially cleaved by the nuclear enzyme Drosha and the cytoplasmic enzyme Dicer to form the functionally mature single-stranded form of the miRNA [53], [54], [55]. We next measured mRNA levels of both Drosha and Dicer in our dorsal and ventral hippocampal tissue samples to better understand the molecular basis of altered mature miR expression levels. Drosha and Dicer mRNA expression levels were measured using qRT-PCR in the untreated animals at early, mid/peri- and late puberty and those levels were compared to animals that were administered our binge EtOH treatment paradigm at mid/peri-puberty. Our results showed a main effect of age on Drosha in both the dorsal and ventral hippocampus (Tables 1, 2; Fig. 4A, B, solid line). Specifically, Drosha mRNA levels were significantly decreased between early and mid/peri-puberty in both regions of the hippocampus. The gene expression levels remained low until late puberty in the dorsal hippocampus, but returned to early pubertal levels in the ventral hippocampus (Fig. 4A, B, solid line), demonstrating region-specific regulation. Further, there was a significant interaction between age and EtOH on Drosha mRNA expression in both hippocampal regions and an overall main effect of EtOH treatment in the ventral hippocampus (Table 1, 2; Fig. 4A, B, dashed line). In both regions, mid/peri-pubertal binge EtOH exposure significantly elevated Drosha mRNA levels immediately following EtOH exposure, suggesting a potential for increased miR biosynthetic processing. These increased levels persisted for up to one month (late puberty, Fig. 4A) following EtOH exposure in the dorsal hippocampus, but were significantly decreased at that same age in the ventral hippocampus (Fig. 4B).

Dicer mRNA expression followed the same pattern as Drosha in untreated animals for both regions of the hippocampus and there was a statistically significant overall main effect of age (Table 1, 2; Fig. 4C, D). Dicer mRNA levels decreased between early and mid/peri-puberty in both regions and the levels remained low until late puberty in the dorsal hippocampus (Fig. 4C). By contrast, at late puberty Dicer mRNA levels were no longer significantly different from those in early puberty in the ventral hippocampus (Fig. 4D). Binge EtOH exposure in mid/peri-puberty had the same effect on Dicer mRNA expression levels in both regions of the hippocampus, with a statistically significant overall main effect of treatment and a significant interaction between age and treatment (Table 1, 2; Fig. 4C, D). Dicer mRNA levels were immediately increased compared to untreated animals following EtOH exposure at mid/peri-puberty, but then by late puberty had decreased significantly to levels even lower than that of untreated animals at early puberty (Fig. 4C, D). In sum, these results demonstrate that both Dicer and Drosha change dynamically throughout pubertal development in the hippocampus and that these levels can be dramatically altered by pubertal binge EtOH exposure (Table 3).

### Binge EtOH exposure alters putative target genes of EtOH-sensitive miRs in the dorsal and ventral hippocampus

**Figure 5:**
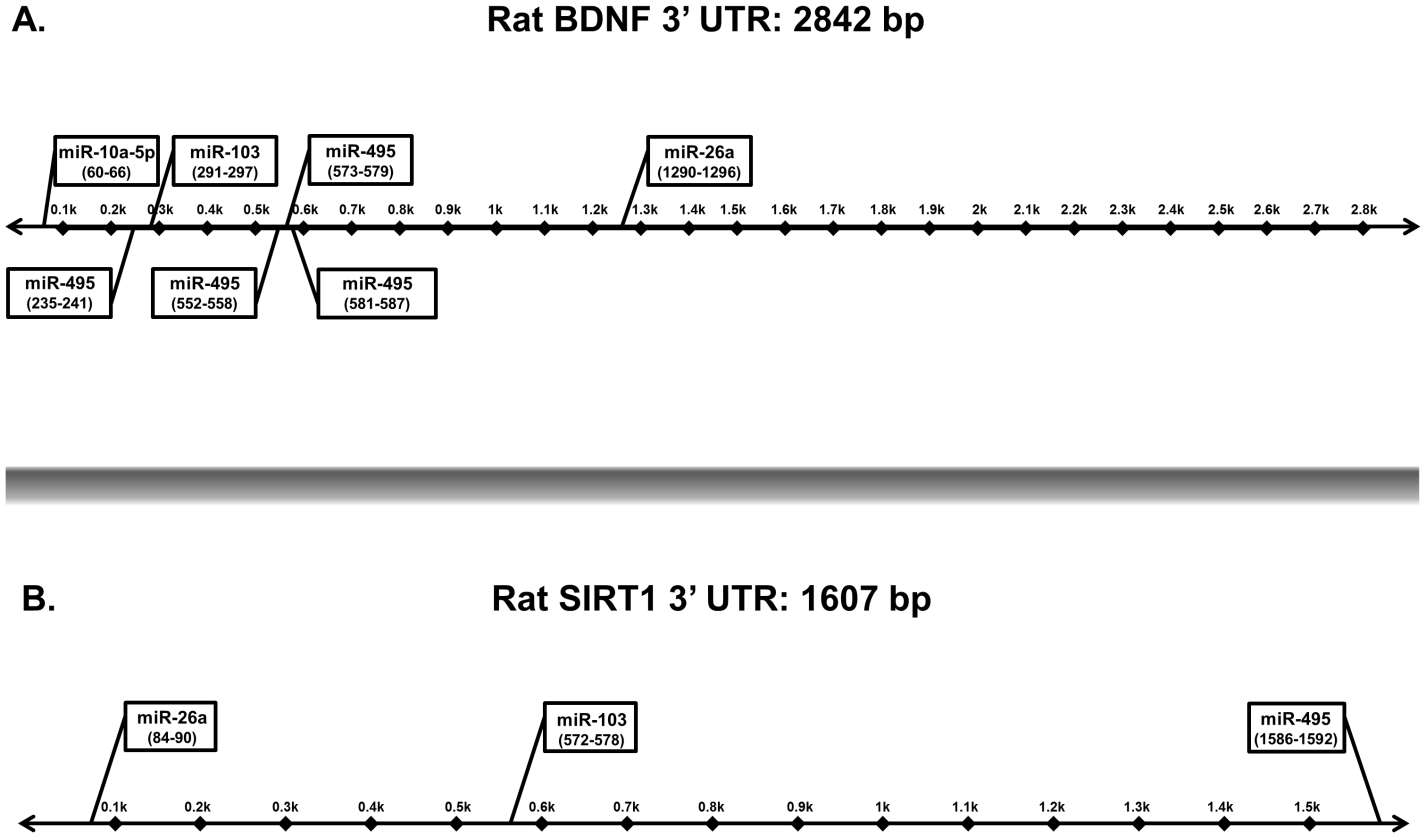
Diagram depicting predicted miR binding sites for BDNF and SIRT1. Schematic diagram of the 3′UTR of (A) BDNF - 2,842 bp and (B) SIRT1- 1,607 bp. The putative binding sites for each miR were predicted using Targetscan (www.targetscan.org) and miRDB (www.miRDB.org) computer algorithm programs. The binding sites were predicted based on the presence of an 8-mer or 7-mer conserved miR seed sequence. Precise seed sequence positions are shown in parentheses.

The most well understood mechanism of miR action is through miR complementary binding to the 3′ untranslated region (UTR) of a primary gene transcript and its subsequent facilitation of mRNA degradation and/or inhibition of mRNA translation [56], [57]. This miR-mediated degradation of mRNA target genes is attributable to observed downstream phenotypic changes. We identified two genes that were putative targets of all 4 EtOH-sensitive miRs in the dorsal and ventral hippocampus using publically available software target prediction programs (Targetscan: www.targetscan.org; miR database: www.miRDB.org) [40], [41], [42]. It is important to note that mammalian miRs bind imperfectly to their 3′UTR targets and therefore, each miR can have multiple gene targets. Putative gene targets can be identified by the presence of a conserved 8 bp seed sequence in the 3′UTR and multiple binding sites within a given target gene can possibly predict the relative specificity of a miR for that gene. Our analysis of potential targets for each EtOH-sensitive miR identified a single putative binding site in the 3′UTR of BDNF for each miR-10a-5p, miR-26a, and miR-103 (Fig. 5). Also, there were 5 possible binding sites on BDNF for miR-495, suggesting miR-495 might have a stronger regulatory effect on BDNF than the other miRs. There were no potential binding sites in the 3′UTR of SIRT1 for miR-10a-5p, but there was a single potential binding site for each of the other miRs tested (miR-26a, miR-103 and miR-495; Fig. 5).

**Figure 6:**
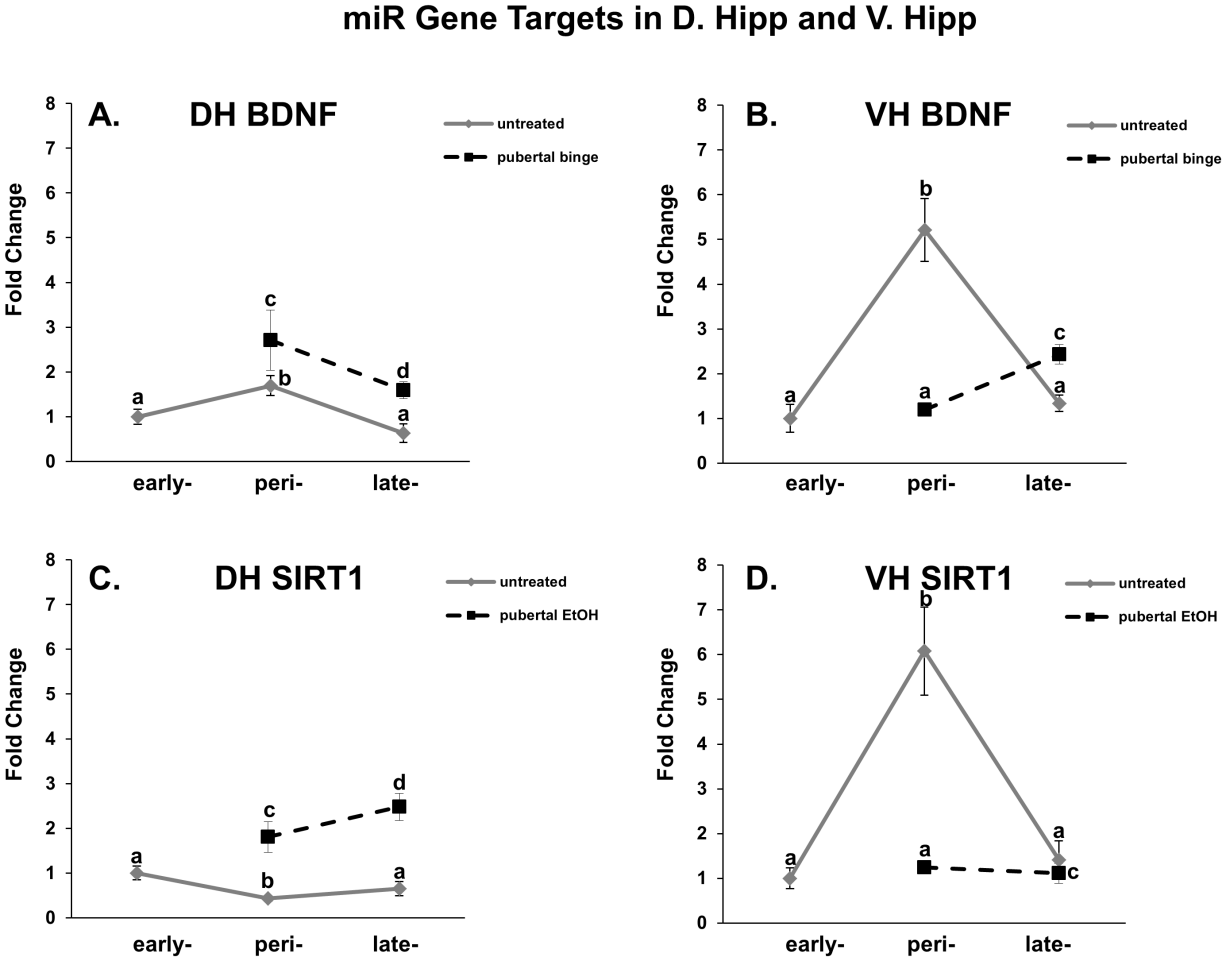
Peri-pubertal binge EtOH exposure differentially alters miR target genes, BDNF and SIRT1, in the dorsal and ventral hippocampus. BDNF (A, C) and SIRT1 (B, D) mRNA levels in the dorsal and ventral hippocampus in untreated (solid line) and EtOH-treated (dashed line) pubertal male rats. N = 10/group. Data represent mean fold change ±SEM as compared to untreated PND 30 animals. Dissimilar letters indicate a statistically significant difference between groups (p<0.05).

To determine whether peri-pubertal binge EtOH exposure altered the normal expression levels of BDNF and SIRT1 mRNA, we compared mRNA levels in untreated animals at each age, early, mid/peri, and late puberty, to animals that had been treated with binge EtOH at mid/peri-puberty in both the dorsal and ventral hippocampus. Overall, there was a significant main effect of age on BDNF mRNA expression in both regions of the hippocampus (Tables 1, 2; Fig. 6A, B, solid line). By contrast, there was a significant main effect of age on SIRT1 mRNA expression in the ventral, but not dorsal, hippocampus (Tables 1, 2; Fig. 6C, D, solid line). There was also a significant main effect of EtOH treatment on BDNF and SIRT1 mRNA expression in both hippocampal regions (Tables 1, 2). A statistically significant interaction between age and EtOH treatment was observed for BDNF and SIRT1 in the dorsal hippocampus (Table 1) and SIRT1 in the ventral hippocampus (Table 2), demonstrating that the effects of binge EtOH exposure on BDNF and SIRT1 mRNA expression was dependent on age. Interestingly, EtOH exposure significantly increased BDNF and SIRT1 mRNA levels compared to untreated animals at mid/peri-puberty in the dorsal hippocampus (Fig. 6A, C, dashed line), while the opposite was observed in the ventral hippocampus (Fig. 6B, D, dashed line). The effects of EtOH persisted for up to one-month post-EtOH exposure for BDNF and SIRT1 in the dorsal hippocampus (Fig. 6A, C) and for BDNF in the ventral hippocampus (Fig. 6A). Although the mRNA levels of SIRT1 were not statistically different from untreated controls one-month following binge EtOH exposure, the data suggest that the normal developmental profile of SIRT1 gene expression was retarded at a pre-pubertal phenotype as a result of mid/peri-pubertal EtOH exposure (Table 3).

### Binge EtOH exposure did not alter circulating testosterone levels

**Figure 7:**
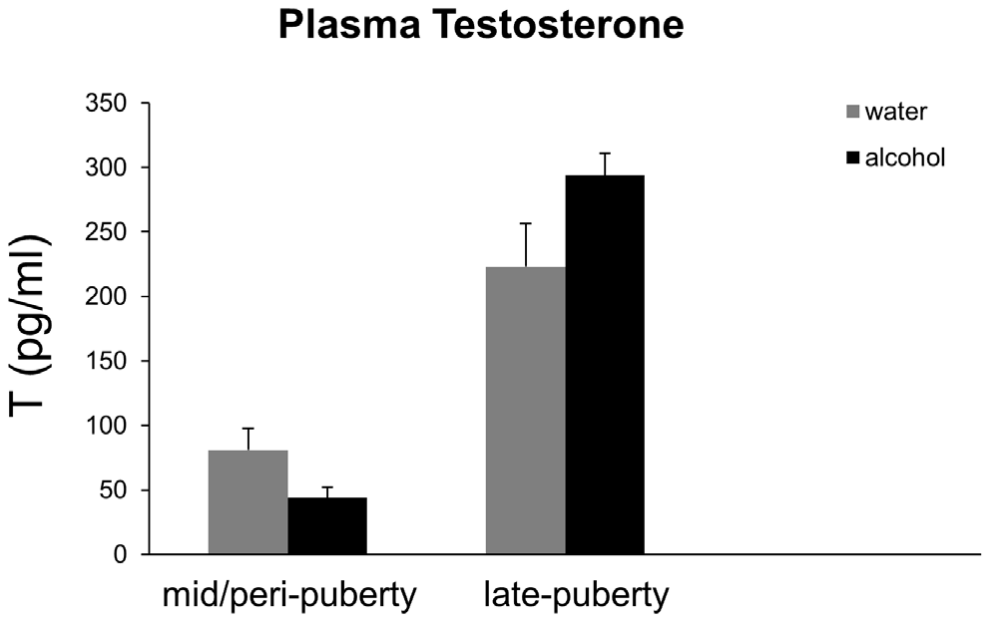
Peri-pubertal binge EtOH exposure did not affect circulating testosterone levels. Plasma concentrations of testosterone (T) 60 min. after the last treatment. Data expressed as mean ±SEM T pg/ml. No statistically significant differences were observed.

Increased gonadal steroid hormones during pubertal development can potentially modulate miR and/or Drosha, Dicer, BDNF and SIRT1 mRNA levels. The animals in this study were kept gonad-intact throughout puberty, however previous studies have demonstrated that EtOH can alter gonadal steroid hormones [12]. To determine the effects of binge EtOH exposure during puberty on circulating gonadal steroid hormone levels in our system, plasma testosterone (T) was measured in each age group on the day of sacrifice. As expected, circulating T levels continued to increase with age in all animals (Fig. 7), demonstrating a normal progression through pubertal development. Mid/peri-pubertal EtOH exposure tended to decrease circulating T levels, but the differences between EtOH-treated animals and controls of the same age group were not statistically significant (Fig. 7). Moreover, there were no apparent long-lasting effects of mid/peri-pubertal EtOH treatment on circulating T levels measured at one-month post EtOH treatment (Fig. 7).

## Discussion

Adolescent alcohol abuse has been shown to exert long-lasting detrimental effects on brain function, neuronal gene expression and behaviors, yet the precise molecular targets of EtOH remain poorly understood. Indeed, previous studies by our laboratory and others have demonstrated both immediate and long-term effects of repeated mid/peri-pubertal binge EtOH exposure on genes that regulate the physiological stress response [12], [14], [15], [17], [58]. Therefore, the goals of this study were to provide a potential mechanistic explanation for EtOH-induced effects on gene expression by quantifying the expression of EtOH sensitive miRs (miR-10a-5p, miR-26a, miR-103 and miR-495) during normal pubertal development in the male rat hippocampus, and then elucidate how peri-pubertal binge EtOH exposure alters the expression of those miRs. Importantly, miRs have emerged as highly conserved critical regulators of downstream gene expression in nearly all physiological systems. Genome-wide miR expression profiles revealed that the miRs investigated in this study, miR-103 and miR-26a, are among the top 15 most abundantly expressed miRs in the rodent hippocampus [59]. Taken together our data revealed that the expression of each miR tested (miR-10a-5p, miR-26a, miR-103 and miR-495) is dynamic across pubertal development and that the developmental profiles for each miR are distinct between the dorsal and ventral hippocampus. Moreover, mid/peri-pubertal binge EtOH exposure altered normal pubertal development expression patterns of miR-10a-5p, miR-26a, miR-103, miR-495, Dicer, Drosha, BDNF and SIRT1 in an age- and brain region-dependent manner. Most striking, our results showed that mid/peri-pubertal binge EtOH exposure had significant long lasting effects on several miRs studied, as well as their processing enzymes and target genes. These effects were evident for as long as one-month following the last EtOH exposure, suggesting that EtOH could have lasting consequences on gene expression profiles in the male rat hippocampus through long-term regulation of miR expression patterns.

Quantifying EtOH-induced changes in Drosha and Dicer mRNA levels can yield insight into the mechanistic actions of EtOH by revealing specific points of EtOH-mediated perturbations along the miR biosynthetic pathway. The biogenesis of mature 22–24 nucleotide (nt), single-stranded miRs involves the following sequential processes: 1) transcription of a 100–1000 nt primary-miR (pri-miR) product, 2) cropping of the transcript by the nuclear RNase III enzyme Drosha into the precursor-miR (pre-miR), 3) cleavage of the pre-miR by the cytoplasmic RNase III enzyme Dicer and 4) loading of the mature miR onto the RNA-induced silencing complex (RISC), which it guides to its mRNA target for degradation or translational inhibition [56], [57]. Notably, the expression of miRNA biogenesis genes (i.e. Drosha, Dicer) was shown to be significantly correlated with addiction-related phenotypes [60]. Our studies revealed the interesting observation that both Drosha and Dicer mRNA significantly decreased between early- and mid/peri-puberty, although these results do not necessarily reflect changes in enzyme catalytic activity. Regardless, decreased mRNA levels of miR biosynthetic enzymes would theoretically result in reduced mature miR levels leading to increased translation of gene targets, consistent with the idea that there are global changes in overall gene expression during adolescent development. The effects of mid/peri-pubertal binge EtOH exposure on Drosha and Dicer mRNA levels continued to persist for as long as one-month after the last EtOH exposure, which raises the possibility of a potential long-term EtOH-induced dysregulation of miR biosynthetic processing.

One of the biggest challenges since the discovery of miRs has been the identification of their target genes. In mammals, their imperfect base pair hybridization with mRNA targets results in promiscuous binding, such that a single miR can have multiple mRNA gene targets. For instance, miR-495 is predicted to target as many as 754 genes in the rat genome (miRDB). Similarly, a single mRNA transcript can be regulated by several different miRs and whether multiple miRs must act in concert to regulate a specific target gene remains unresolved. Indeed, BDNF is predicted to be targeted by 51 miRs (miRDB), therefore the results shown herein are not exhaustive of all potential regulators of BDNF. Nevertheless, our data demonstrating differential expression of the same miR in functionally distinct hippocampal regions strongly implied that the targets of these miRs were also differentially expressed. Therefore, we identified putative target genes of miR-10a-5p, miR-26a, miR-103 and miR-495 that were relevant to known dorsal and ventral hippocampus functions using target prediction software programs, Targetscan and MirDB. For instance, BDNF was identified as a putative target gene for miR-10a-5p, miR-26a, miR-103 and miR-495. BDNF plays a fundamental role in guiding neurodevelopment as well as in the fine-tuning of synaptic plasticity, a critical event during adolescent brain development. Consistent with our hypothesis, decreased expression of miR-26a and miR-495 correlated with increased BDNF gene expression in the dorsal hippocampus immediately following mid/peri-pubertal binge EtOH exposure and this increase remained significantly elevated up to one-month post EtOH exposure. Previous studies have also shown that regulation of BDNF expression is mediated by miR-26a targeting the conserved BDNF 3′UTR sequence [61]. Importantly, both BDNF gene variants and miR-26a expression have been implicated in the vulnerability and onset of schizophrenia, alcohol abuse and mood disorders in both human patients and rodent models [61], [62], [63], [64], [65], [66]. Therefore, mid/peri-pubertal disruption of miR-26a and miR-495 expression by binge EtOH exposure could result in altered BDNF expression throughout pubertal development.

The histone deacetylase sirtuin 1 (SIRT1) was also predicted by computer algorithms to be a putative gene target of miR-26a, mir-103 and miR-495. Our data demonstrated that mid/peri-pubertal binge EtOH exposure had long-lasting effects on SIRT1 gene expression, showing significantly increased mRNA levels lasting until late puberty in the dorsal hippocampus. Similar to BDNF, SIRT1 has recently been implicated as critical for mediating synaptic plasticity, one mechanism underlying memory formation in rodent and human cell models [67], [68]. Moreover, many studies have demonstrated that EtOH exposure has long-lasting consequences on gene expression, which may be regulated, in part, by miRs targeting chromatin-modifying enzymes, such as SIRT1.

Overall, our data reveal novel findings about the age and brain-region specific expression of miR-10a-5p, miR-26a, miR-103, and miR-495 during pubertal development in male rats. Further, we showed that mid/peri-pubertal binge EtOH exposure significantly alters the normal expression profile of these miRs, their biosynthetic processing enzymes, and two of their putative target genes, BDNF and SIRT1. However, it is important to note that these results are not necessarily predictive of miRs in females or other species, as several studies have demonstrated both species and sex-specific expression profiles for miRs [69], [70], [71]. Moreover, the precise molecular targets of EtOH in the biogenesis of miRs remain unclear and require further investigation. An important next step is the identification of specific cell types (i.e. neurons vs. glia) in which miRs are affected by binge EtOH exposure, as our study was limited to whole hippocampal tissue homogenates. Taken together, our data raise the possibility that EtOH modulation of these fours miRs is a potential cellular mechanism underlying long-term changes in gene expression induced by adolescent EtOH abuse.
